# A generalizable relationship between mortality and time-to-death among breast cancer patients can be explained by tumour dormancy

**DOI:** 10.1007/s10549-019-05334-5

**Published:** 2019-07-01

**Authors:** Vasily Giannakeas, Steven A. Narod

**Affiliations:** 1 Women’s College Research Institute, 76 Grenville Street, Toronto, ON M5S 1B1 Canada; 2 Dalla Lana School of Public Health, University of Toronto, Toronto, Canada

**Keywords:** Breast cancer, Tumor dormancy, Survival

## Abstract

**Background:**

Women with ER-positive breast cancer may recur as late as 20 years post-diagnosis. The reason for this delayed recurrence is unknown. We studied survival patterns, including time-to-death in 123,705 women with stage I to III invasive breast cancer, enrolled in the SEER database. Among these 76.8% were ER-positive and 23.2% were ER-negative.

**Methods:**

We divided the cohort into ten classes with varying risks of death from breast cancer. The 20-year mortality for women in the highest risk decile 10 was 69% versus 5% for women in the lowest decile 1. The difference in the time-to-death by decile could be explained by a variable *α* which represents the annual rate of reactivation from tumour dormancy.

**Results:**

The duration of tumour dormancy was much longer, on average, for ER-positive breast cancers than for ER-negative breast cancers. Reactivation from tumour dormancy appears to occur at random and may explain the very long time to cancer recurrence in women with small node-negative ER-positive breast cancers.

**Conclusion:**

The clinical course of women with low-risk ER-positive breast cancer is inherently unpredictable and consequently death is equally as likely to occur at year 3 than at year 20.

**Electronic supplementary material:**

The online version of this article (10.1007/s10549-019-05334-5) contains supplementary material, which is available to authorized users.

## Introduction

In nearly all cases, a woman who dies of breast cancer experiences a distant recurrence prior to her death. There are several steps between diagnosis and death—initially cancer cells must migrate from the breast to a distant site and establish a stable presence. Second, the cancer must proliferate in the metastatic niche or set up secondary metastases. The cancer must survive eradication by hormonal therapy and/or chemotherapy and be recalcitrant to a host immune response. One can also posit a dormant period wherein the cancer cells are stable in the metastatic niche, but are not proliferating; in this case, transition from a dormant to an active state is a further condition of fatality. The time from diagnosis to death varies from months to years and may be particularly long for women with ER-positive breast cancers [1]. It has been proposed that the long survival times for women with ER-positive cancers are the consequence of a prolonged dormant period, but this is unproven [1].

Previously, we used three complementary metrics to describe the time course of deaths in various groups of women with breast cancer [2]. The first is the annual mortality rate post-diagnosis, the second is the twenty-year Kaplan–Meier actuarial survival, and the third is the frequency distribution of times to death. Using these metrics, we described the heterogeneous nature of time-to-death according to patient subgroup and tumour sub-type [2].

Women with triple-negative breast cancers experience a peak distant recurrence rate at 1 year (15% per year) followed by a sharp decline that falls below that of ER-positive breast cancers at 5 years [3, 4]. The mortality rate for ER-positive breast cancer patients is stable between year 3 and year 20 [3]. If a woman has a small node-negative ER-positive breast cancer she is equally as likely to die in year 20 as in year three [2]. Unpredictability in time-to-death is an inherent property common to all breast cancer subgroups with low fatality rates [2]. We are able to calculate life expectancy at a *group* level but are unable to predict date of death at an *individual* level. We propose that variation in time-to-death can be explained by reference to the rate of transition of metastases from a dormant to an active state. We sought to determine whether tumour dormancy is a feature of all breast cancer cases or only those which are ER-positive.

## Methods

### Study subjects

We extracted data on all breast cancer cases from the Surveillance, Epidemiology and End Results (SEER) data set diagnosed from 1990 to 1999. Patient characteristics include year of birth, year of diagnosis, age at diagnosis, race and household income. Tumour characteristics include grade (well differentiated—I, moderately differentiated—II, poorly differentiated—III, undifferentiated/anaplastic—IV, unknown), tumour size, nodal status (N0, N1, N2, N3, unknown), stage (I, II, III), estrogen receptor (ER) (negative, positive) and progesterone receptor (PR) status (negative, positive, unknown). Cancer treatment variables include radiation (no, yes, unknown) and chemotherapy (no/unknown, yes). Information on tamoxifen use and on surgical procedures for the primary tumour were not available in SEER prior to 1998.

We excluded patients with a prior history of cancer, patients with DCIS, stage IV breast cancer or unknown stage. We excluded patients whose tumour size or estrogen receptor status was unknown. The final cohort consisted of 123,705 women with a first primary invasive breast cancer. The mean follow-up was 13.1 years and among alive patients 95% were followed 15 years or more. Patients were followed from breast cancer diagnosis to breast cancer-specific death, other cause-of-death, loss to follow-up, or 20 years post-diagnosis. The patient cohort is described in Table 1.

**Table 1:** Baseline characteristics of breast cancer patient cohort by ER status

Descriptor	Value	Total	ER-positive	ER-negative
Total		123,705	94,944 (76.8%)	28,761 (23.2%)
Age at diagnosis (cont.)	Mean (SD)	60.1 (14.1)	61.5 (13.8)	55.4 (14.1)
	Median (IQR)	60.0 (49.0–71.0)	62.0 (51.0–72.0)	54.0 (45.0–66.0)
Age at diagnosis (cat.)	< 40	8549 (6.9%)	4901 (5.2%)	3648 (12.7%)
	40–49	24,036 (19.4%)	16,564 (17.4%)	7472 (26.0%)
	50–59	27,544 (22.3%)	20,578 (21.7%)	6966 (24.2%)
	60–69	27,825 (22.5%)	22,560 (23.8%)	5265 (18.3%)
	70–79	24,942 (20.2%)	21,119 (22.2%)	3823 (13.3%)
	80+	10,809 (8.7%)	9222 (9.7%)	1587 (5.5%)
Ethnicity	White	104,308 (84.3%)	81,667 (86.0%)	22,641 (78.7%)
	Black	9406 (7.6%)	5711 (6.0%)	3695 (12.8%)
	East Asian	4777 (3.9%)	3688 (3.9%)	1089 (3.8%)
	Southeast Asian	2898 (2.3%)	2112 (2.2%)	786 (2.7%)
	Other/unknown	2316 (1.9%)	1766 (1.9%)	550 (1.9%)
Neighborhood household income (cont.)	Mean (SD)	35,623 (7102)	35,683 (7137)	35,426 (6981)
	Median (IQR)	34,970 (30,150–38,930)	34,970 (30,150–38,930)	34,970 (30,150–38,830)
Neighborhood household income (cat.)	< 30,000	28,960 (23.4%)	21,989 (23.2%)	6971 (24.2%)
	30,000–35,000	35,818 (29.0%)	27,399 (28.9%)	8419 (29.3%)
	35,000–40,000	29,135 (23.6%)	22,331 (23.5%)	6804 (23.7%)
	40,000+	29,792 (24.1%)	23,225 (24.5%)	6567 (22.8%)
Stage	I	61,022 (49.3%)	49,902 (52.6%)	11,120 (38.7%)
	II	44,835 (36.2%)	32,451 (34.2%)	12,384 (43.1%)
	III	17,848 (14.4%)	12,591 (13.3%)	5257 (18.3%)
Tumour grade	I	16,823 (13.6%)	15,740 (16.6%)	1083 (3.8%)
	II	43,791 (35.4%)	38,069 (40.1%)	5722 (19.9%)
	III	38,831 (31.4%)	22,411 (23.6%)	16,420 (57.1%)
	IV	3335 (2.7%)	1914 (2.0%)	1421 (4.9%)
	Unknown	20,925 (16.9%)	16,810 (17.7%)	4115 (14.3%)
Tumour size (cont.)	Mean (SD)	2.2 (1.8)	2.1 (1.7)	2.6 (2.0)
	Median (IQR)	1.8 (1.2–2.5)	1.6 (1.1–2.5)	2.0 (1.4–3.0)
Tumour size (cat.)	< 1 cm	17,718 (14.3%)	14,903 (15.7%)	2815 (9.8%)
	1–2 cm	50,190 (40.6%)	40,667 (42.8%)	9523 (33.1%)
	2–3 cm	29,466 (23.8%)	21,713 (22.9%)	7753 (27.0%)
	3–5 cm	18,034 (14.6%)	12,254 (12.9%)	5780 (20.1%)
	5+ cm	8297 (6.7%)	5407 (5.7%)	2890 (10.0%)
Nodal involvement	N0	82,717 (66.9%)	64,660 (68.1%)	18,057 (62.8%)
	N1	25,750 (20.8%)	19,419 (20.5%)	6331 (22.0%)
	N2	9516 (7.7%)	6906 (7.3%)	2610 (9.1%)
	N3	5374 (4.3%)	3733 (3.9%)	1641 (5.7%)
	Unknown	348 (0.3%)	226 (0.2%)	122 (0.4%)
PR status	Negative	38,567 (31.2%)	14,749 (15.5%)	23,818 (82.8%)
	Positive	80,481 (65.1%)	76,390 (80.5%)	4091 (14.2%)
	Unknown	4657 (3.8%)	3805 (4.0%)	852 (3.0%)
Chemotherapy	No/Unknown	82,377 (66.6%)	69,069 (72.7%)	13,308 (46.3%)
	Yes	41,328 (33.4%)	25,875 (27.3%)	15,453 (53.7%)
Radiotherapy	No	64,173 (51.9%)	48,954 (51.6%)	15,219 (52.9%)
	Yes	57,138 (46.2%)	44,367 (46.7%)	12,771 (44.4%)
	Unknown	2394 (1.9%)	1623 (1.7%)	771 (2.7%)
Vital status at 20 years post-diagnosis	Alive	59,739 (48.3%)	45,233 (47.6%)	14,506 (50.4%)
	Breast	23,653 (19.1%)	16,151 (17.0%)	7502 (26.1%)
	Other COD	33,180 (26.8%)	27,596 (29.1%)	5584 (19.4%)
	Unknown COD	7133 (5.8%)	5964 (6.3%)	1169 (4.1%)

*SD* standard deviation, *IQR* interquartile range, *COD* cause of death

### Classifying probability of breast cancer death

We aimed to delineate a statistical relationship between the probability of death from breast cancer and time-to-death. The overall strategy was to generate a series of mortality curves for patient subgroups with varying risk profiles, defined by tumour and host factors. The probability of death from breast cancer was defined as the actuarial risk of dying of breast cancer by 20 years post-diagnosis.

Using all the available data, ten risk groups of equal size were constructed and ranked on their 20-year actuarial probability of death from the lowest risk (decile 1) to highest risk (decile 10). Survival was modelled using a disease risk score approach in combination with Cox regression analysis, with year at diagnosis, age, income, race, tumour size, stage, grade, nodal status, ER status, PR status, radiotherapy and chemotherapy as predictors. To account for potential nonlinearity of continuous variables, we modelled natural cubic splines for age at diagnosis, income and tumour size. The Breslow estimator was used to obtain cumulative baseline hazard functions and generate an actuarial probability of death from breast cancer at 20 years for each patient [5].

### Modelling tumour dormancy

We asked if the differences in the mortality experience of the ten risk groups could be explained entirely by a model where the duration of tumour dormancy varied. Under this model, after reactivation from the dormant state, all cancers experience the same growth rate. We model dormancy as a single stochastic rate from dormant to active. We assume that all women in the same risk decile experience the same rate of reactivation, that reactivation occurs at random and that the annual reactivation rate is constant for the entire 20-year follow-up period. We define the tumour reactivation factor *α* as a scalar variable that represents the *rate* of cancer reactivation. The factor is assumed to be independent of time from diagnosis and is modelled as a Poisson process. The tumour reactivation factor can take on any value greater than zero; where an *α* value that approaches zero results in a low tumour reactivation, and an *α* value that approaches infinity results in an immediate reactivation. Quantitatively, the tumour reactivation factor represents the rate of cancers becoming reactivated per year of follow-up. For example, an *α* of 0.10 [year^−1^] would result in 10,000 of 100,000 cancers reactivated in any given year. This corresponds to a mean reactivation time of 10 years (1/*α*), with 63.2% of cancers being reactivated within 10 years and 86.5% reactivated within 20 years ($1 - {\text{e}}^{{\left( { - 0.1 \times 20} \right)}}$). We used the highest risk decile (decile 10) as the reference distribution to model tumour dormancy in the remaining deciles. We assumed that the women in the highest risk decile did not experience a period of tumour dormancy and that patients in risk deciles 9 to 1 experience tumour dormancy to an increasingly greater degree. Each cancer was initially dormant and then might or might not have been activated over the 20 year follow-up period. Normalized mortality rate distributions were generated for each value *α*, ranging from 0.01 to 10.0, by 0.01 increments, using decile 10 as the baseline reference group (no dormant period).^1^ We then sought to identify, for each decile, the single value of *α* which generated a theoretical normalized mortality rate curve which most closely corresponded to the empiric distribution. To measure goodness of fit, the Mean Square Error (MSE) for each *α* value was calculated, by comparing to the true distribution observed in each decile.

Next we aimed to identify a fitted value *c* for each decile which when multiplied by the normalized mortality rates will result in the observed 20-year breast cancer specific mortality. The multiplication factor (*c*) in combination with the tumour reactivation factor (*α*) are used to generate predicted mortality rates, Kaplan–Meier curves, and distributions of time-to-death. Fitted values for *c* were estimated using an iterative approach similar to what was done for *α* estimation.

### Identifying predictors of tumour dormancy

After exploring the relationship between risk of breast cancer death and tumour dormancy, we sought to determine independent predictors of tumour dormancy. To do this, we performed quantile regression to model the median time-to-death among women that died from breast cancer. Predictors of time-to-death include year of diagnosis, age, ethnicity, tumour grade, nodal status, ER status, PR status, radiotherapy and chemotherapy. Inverse probability of censor weights was incorporated into the quantile regression to account for differences in follow-up time between subjects (see supplemental methods).

## Results

**Fig. 1:**
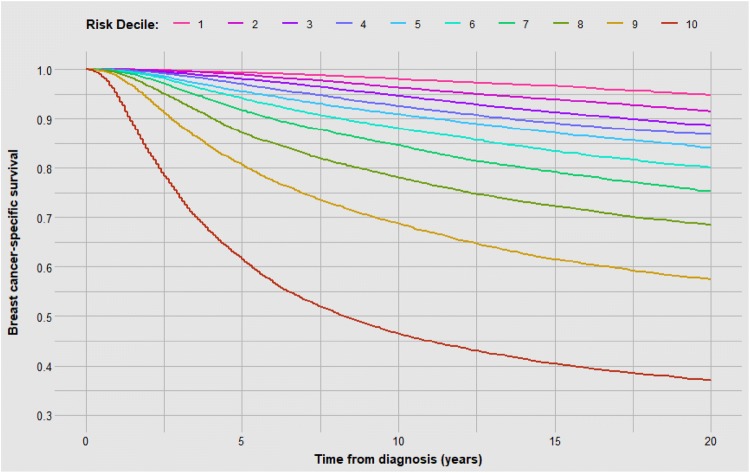
Kaplan–Meier curves observed for each risk decile of breast cancer patients

We divided 123,705 women into ten risk deciles based on the variables in the SEER database. The patient and tumour characteristics for each of the ten risk groups are shown in Table 2. The mean probability of death (mortality) for the 12,370 women in the lowest decile was 5.1% and for the 12,370 women in the highest decile was 69.2%. The 20-year Kaplan–Meier survival curves are presented in Fig. 1.

**Table 2:** Baseline characteristics of breast cancer cohort by decile of risk

Variable	Value	1	2	3	4	5	6	7	8	9	10
Total	123,705	12,370 (10.0%)	12,371 (10.0%)	12,370 (10.0%)	12,371 (10.0%)	12,370 (10.0%)	12,371 (10.0%)	12,371 (10.0%)	12,370 (10.0%)	12,371 (10.0%)	12,370 (10.0%)
Age at diagnosis (cont.)	Mean (SD)	59.6 (10.7)	57.9 (11.4)	59.8 (11.9)	61.9 (13.0)	62.7 (14.7)	61.6 (14.9)	58.6 (14.3)	59.3 (15.4)	59.7 (16.2)	60.0 (16.4)
Ethnicity	White	10,448 (84.5%)	10,905 (88.1%)	11,092 (89.7%)	10,909 (88.2%)	10,572 (85.5%)	10,184 (82.3%)	10,654 (86.1%)	10,369 (83.8%)	9657 (78.1%)	9518 (76.9%)
	Black	234 (1.9%)	181 (1.5%)	314 (2.5%)	629 (5.1%)	959 (7.8%)	1110 (9.0%)	790 (6.4%)	1218 (9.8%)	1922 (15.5%)	2049 (16.6%)
	East Asian	1071 (8.7%)	734 (5.9%)	510 (4.1%)	390 (3.2%)	371 (3.0%)	511 (4.1%)	384 (3.1%)	267 (2.2%)	294 (2.4%)	245 (2.0%)
	Southeast Asian	265 (2.1%)	352 (2.8%)	266 (2.2%)	244 (2.0%)	234 (1.9%)	325 (2.6%)	349 (2.8%)	294 (2.4%)	272 (2.2%)	297 (2.4%)
	Other/unknown	352 (2.8%)	199 (1.6%)	188 (1.5%)	199 (1.6%)	234 (1.9%)	241 (1.9%)	194 (1.6%)	222 (1.8%)	226 (1.8%)	261 (2.1%)
Neighbourhood household income (cont.)	Mean (SD)	36,621 (6850)	36,796 (7018)	35,888 (7058)	35,547 (7163)	35,271 (7231)	35,556 (7158)	35,493 (7066)	35,129 (7061)	35,005 (7046)	34,929 (7102)
Stage	I	12,299 (99.4%)	11,922 (96.4%)	11,710 (94.7%)	10,925 (88.3%)	8820 (71.3%)	4188 (33.9%)	998 (8.1%)	144 (1.2%)	16 (0.1%)	0 (0.0%)
	II	66 (0.5%)	449 (3.6%)	657 (5.3%)	1442 (11.7%)	3526 (28.5%)	8055 (65.1%)	11,089 (89.6%)	11,435 (92.4%)	7280 (58.8%)	836 (6.8%)
	III	5 (0.0%)	0 (0.0%)	3 (0.0%)	4 (0.0%)	24 (0.2%)	128 (1.0%)	284 (2.3%)	791 (6.4%)	5075 (41.0%)	11,534 (93.2%)
Tumour grade	I	10,363 (83.8%)	1894 (15.3%)	977 (7.9%)	1178 (9.5%)	1004 (8.1%)	617 (5.0%)	341 (2.8%)	238 (1.9%)	171 (1.4%)	40 (0.3%)
	II	1676 (13.5%)	7784 (62.9%)	6139 (49.6%)	4654 (37.6%)	4322 (34.9%)	5046 (40.8%)	4741 (38.3%)	3475 (28.1%)	3470 (28.0%)	2484 (20.1%)
	III	21 (0.2%)	440 (3.6%)	2036 (16.5%)	3514 (28.4%)	4382 (35.4%)	4015 (32.5%)	4510 (36.5%)	6094 (49.3%)	6346 (51.3%)	7473 (60.4%)
	IV	6 (0.0%)	90 (0.7%)	245 (2.0%)	322 (2.6%)	361 (2.9%)	341 (2.8%)	436 (3.5%)	478 (3.9%)	497 (4.0%)	559 (4.5%)
	Unknown	304 (2.5%)	2163 (17.5%)	2973 (24.0%)	2703 (21.8%)	2301 (18.6%)	2352 (19.0%)	2343 (18.9%)	2085 (16.9%)	1887 (15.3%)	1814 (14.7%)
Tumour size (cont.)	Mean (SD)	1.1 (2.4)	1.2 (0.5)	1.3 (0.6)	1.4 (0.6)	1.7 (0.8)	2.2 (1.0)	2.5 (1.2)	2.8 (1.4)	3.1 (1.8)	4.4 (3.0)
Nodal involvement	N0	12,364 (100.0%)	12,341 (99.8%)	12,113 (97.9%)	11,656 (94.2%)	11,536 (93.3%)	9511 (76.9%)	6480 (52.4%)	4538 (36.7%)	1932 (15.6%)	246 (2.0%)
	N1–N3	6 (0.0%)	30 (0.2%)	257 (2.1%)	715 (5.8%)	834 (6.7%)	2860 (23.1%)	5891 (47.6%)	7830 (63.3%)	10,424 (84.3%)	11,793 (95.3%)
	Unknown	0 (0.0%)	0 (0.0%)	0 (0.0%)	0 (0.0%)	0 (0.0%)	0 (0.0%)	0 (0.0%)	2 (0.0%)	15 (0.1%)	331 (2.7%)
ER status	Negative	487 (3.9%)	470 (3.8%)	1126 (9.1%)	2750 (22.2%)	3863 (31.2%)	2895 (23.4%)	3234 (26.1%)	4283 (34.6%)	4539 (36.7%)	5114 (41.3%)
	Positive	11,883 (96.1%)	11,901 (96.2%)	11,244 (90.9%)	9621 (77.8%)	8507 (68.8%)	9476 (76.6%)	9137 (73.9%)	8087 (65.4%)	7832 (63.3%)	7256 (58.7%)
PR status	Negative	1386 (11.2%)	1112 (9.0%)	2103 (17.0%)	3904 (31.6%)	4930 (39.9%)	3877 (31.3%)	4164 (33.7%)	5354 (43.3%)	5478 (44.3%)	6259 (50.6%)
	Positive	10,423 (84.3%)	10,771 (87.1%)	9744 (78.8%)	7925 (64.1%)	6957 (56.2%)	8055 (65.1%)	7748 (62.6%)	6629 (53.6%)	6472 (52.3%)	5757 (46.5%)
	Unknown	561 (4.5%)	488 (3.9%)	523 (4.2%)	542 (4.4%)	483 (3.9%)	439 (3.5%)	459 (3.7%)	387 (3.1%)	421 (3.4%)	354 (2.9%)
Chemotherapy	No/Unknown	11,509 (93.0%)	10,553 (85.3%)	10,356 (83.7%)	9671 (78.2%)	8983 (72.6%)	7973 (64.4%)	6696 (54.1%)	6267 (50.7%)	5578 (45.1%)	4791 (38.7%)
	Yes	861 (7.0%)	1818 (14.7%)	2014 (16.3%)	2700 (21.8%)	3387 (27.4%)	4398 (35.6%)	5675 (45.9%)	6103 (49.3%)	6793 (54.9%)	7579 (61.3%)
Radiotherapy	No	3698 (29.9%)	3830 (31.0%)	5617 (45.4%)	6546 (52.9%)	7314 (59.1%)	7138 (57.7%)	7233 (58.5%)	7957 (64.3%)	7625 (61.6%)	7215 (58.3%)
	Yes	8552 (69.1%)	8383 (67.8%)	6621 (53.5%)	5639 (45.6%)	4846 (39.2%)	5012 (40.5%)	4862 (39.3%)	4132 (33.4%)	4387 (35.5%)	4704 (38.0%)
	Unknown	120 (1.0%)	158 (1.3%)	132 (1.1%)	186 (1.5%)	210 (1.7%)	221 (1.8%)	276 (2.2%)	281 (2.3%)	359 (2.9%)	451 (3.6%)
Vital status at 20 years post-diagnosis	Alive	8527 (68.9%)	8453 (68.3%)	7366 (59.5%)	6198 (50.1%)	5626 (45.5%)	5791 (46.8%)	6051 (48.9%)	5037 (40.7%)	4092 (33.1%)	2598 (21.0%)
	Breast	462 (3.7%)	813 (6.6%)	1116 (9.0%)	1297 (10.5%)	1491 (12.1%)	1885 (15.2%)	2476 (20.0%)	3190 (25.8%)	4301 (34.8%)	6622 (53.5%)
	Other COD	2730 (22.1%)	2539 (20.5%)	3185 (25.7%)	4032 (32.6%)	4364 (35.3%)	3858 (31.2%)	3153 (25.5%)	3403 (27.5%)	3283 (26.5%)	2633 (21.3%)
	Unknown COD	651 (5.3%)	566 (4.6%)	703 (5.7%)	844 (6.8%)	889 (7.2%)	837 (6.8%)	691 (5.6%)	740 (6.0%)	695 (5.6%)	517 (4.2%)
Probability of BC death at 20 years	Mean (SD)	5.14% (1.08%)	8.09% (0.57%)	9.98% (0.56%)	12.12% (0.71%)	15.04% (1.02%)	19.41% (1.46%)	24.75% (1.63%)	31.59% (2.41%)	43.39% (4.81%)	69.19% (11.60%)

*SD* standard deviation, *IQR* interquartile range, *COD* cause of death

**Fig. 2:**
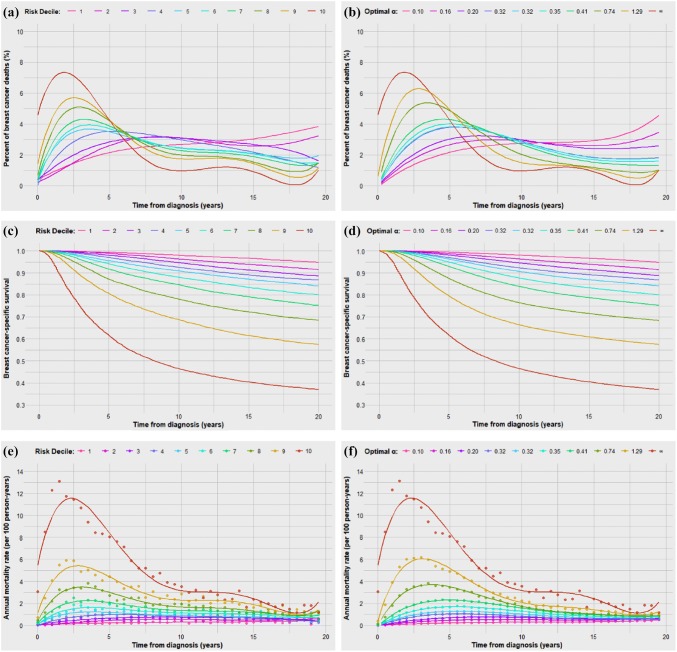
Observed and predicted survival metrics for each decile using optimal *α* and *c* value. Time-to-death distributions for observed (**a**) and predicted (**b**) deciles, Kaplan–Meier survival curves for observed (**c**) and predicted (**d**) deciles, and biannual mortality rates and distributions for observed (**e**) and predicted (**f**) deciles

We sought to generate survival curves using the dormancy model, assuming patients in decile 10 (the reference group) experienced no dormancy and all other deciles varied only in the duration of tumour dormancy. That is, the differences in survival curves between risk groups depended only on differences in the tumour reactivation factor (*α*) and multiplier (*c*). Details on the generation of survival curves and distributions are available in the supplemental methods. We compared the observed and modelled curves for time-to-death for each of the ten deciles. The fit was good by inspection (Fig. 2a, b).

Using *α* and *c*, we generated modelled curves for annual mortality and Kaplan–Meier survival and compared these with the actual curves for the ten deciles (Figs. 2c, d and 2e, f). The fitted values for *α* and *c* for each decile are presented in Table 3. The relationship between tumour dormancy factor (*α*), *c* value and 20-year breast cancer mortality across the nine deciles is presented graphically in eFigure 1.

**Table 3:** Factors related to breast cancer mortality and time-to-death in risk subgroups (deciles)

Risk Decile	Number of patients (*N*)	Annual death rate (per 100 person-years)	20-year actuarial mortality (%)	Peak mortality time (years)^a^	Median time-to-death (years)	10th percentile time-to-death (years)	90th percentile time-to-death (years)	*α* (reactivations per person-year)	*C* value
1	12,370	0.25	5.3	19.0	12.2	4.0	18.7	0.10	0.0542
2	12,371	0.43	8.6	19.5	11.0	4.3	18.4	0.16	0.0898
3	12,370	0.60	11.3	9.5	10.3	3.5	17.6	0.20	0.1202
4	12,371	0.74	13.2	5.5	8.9	3.0	16.7	0.32	0.1418
5	12,370	0.90	16.0	3.5	8.6	2.6	17.4	0.32	0.1739
6	12,371	1.17	20.0	4.0	8.1	2.6	16.9	0.35	0.2234
7	12,371	1.51	24.8	3.0	7.6	2.3	16.7	0.41	0.2845
8	12,370	2.12	31.7	3.0	6.4	1.9	15.9	0.74	0.3810
9	12,371	3.22	42.5	2.5	5.6	1.7	14.8	1.29	0.5541
10	12,370	6.64	63.0	2.0	3.8	1.1	12.3	InF (Ref.)	0.9934

^a^Peak mortality rate from biannual mortality rate curves (Fig. 2e)

We conducted an analysis using a quantile regression model which sought to identify factors which predicted time-to-death. The outcome was change in median time-to-death for a subgroup of breast cancer patients. The results of the regression model are presented in Table 4. The median time to death among all breast cancers was 5.67 years. On average, women with ER-positive breast cancers had a median time-to-death 1.50 years longer this. Women with ER-negative breast cancers had a median time to death 2.17 years shorter. The main effect of ER-status was attenuated after adjusting for other risk factors correlated with ER status. In the multivariable analysis, independent predictors of time-to-death include ethnicity, tumour size, grade, nodal status and PR-status (Table 4). Neither radiotherapy nor chemotherapy was a clinically significant predictor of median time-to-death.

**Table 4:** Predictors of time-to-death in breast cancer patients

Predictor	Value	Unadjusted		Adjusted	
		Median time-to-death (years)	Difference (years)^a^	Difference (years)^b^	*P*
Overall/Reference		5.67		13.05^c^	< 0.0001
Year of diagnosis	1990	5.42		Reference	
	1991	5.67	0.25	0.17	0.3609
	1992	5.67	0.25	0.22	0.2899
	1993	5.58	0.17	0.35	0.0599
	1994	6.00	0.58	0.53	0.0054
Age at diagnosis	< 50	5.58		Reference	
	50–59	5.92	0.33	0.07	0.5807
	60–69	6.17	0.58	− 0.06	0.6551
	70–80	5.25	− 0.33	− 1.18	< 0.0001
Ethnicity	White	5.75		Reference	
	Black	4.33	− 1.42	− 0.32	0.009
	East Asian	6.67	0.92	0.93	0.0058
	Southeast Asian	6.08	0.33	0.09	0.8208
	Other/unknown	6.17	0.42	0.42	0.4176
Tumour size (cm)	< 1 cm	8.33		Reference	
	1–2 cm	7.33	− 1.00	− 0.65	0.0792
	2–3 cm	5.58	− 2.75	− 1.42	< 0.0001
	3–5 cm	4.75	− 3.58	− 1.76	< 0.0001
	5+ cm	3.83	− 4.50	− 2.16	< 0.0001
Tumour grade	I	11.17		Reference	
	II	7.58	− 3.58	− 2.82	< 0.0001
	III	4.25	− 6.92	− 4.78	< 0.0001
	IV	3.92	− 7.25	− 5.18	< 0.0001
	Unknown	6.25	− 4.92	− 3.57	< 0.0001
Nodal status	N0	6.83		Reference	
	N1	5.67	− 1.17	− 0.69	< 0.0001
	N2	5.00	− 1.83	− 1.08	< 0.0001
	N3	3.83	− 3.00	− 2.03	< 0.0001
	Unknown	2.42	− 4.42	− 2.32	< 0.0001
ER status	Positive	7.17		Reference	
	Negative	3.50	− 3.67	− 1.76	< 0.0001
PR status	Positive	7.25		Reference	
	Negative	3.83	− 3.42	− 1.7	< 0.0001
	Unknown	5.08	− 2.17	− 1.35	< 0.0001
Radiotherapy	No	5.42		Reference	
	Yes	6.08	0.67	0.26	0.0214
	Unknown	5.08	− 0.33	− 0.47	0.1043
Chemotherapy	No/unknown	6.58		Reference	
	Yes	5.17	− 1.42	− 0.31	0.0091

^a^Difference in median time-to-death relative to reference value

^b^Independent difference in median time-to-death after adjusting for all covariates in table

^c^Median time-to-death of the reference group; diagnosed in year 1990, age < 50, white ethnicity, < 1 cm, grade I, N0, ER-positive, PR-positive, no radiotherapy, no/unknown chemotherapy

### Stratification by estrogen receptor status

**Fig. 3:**
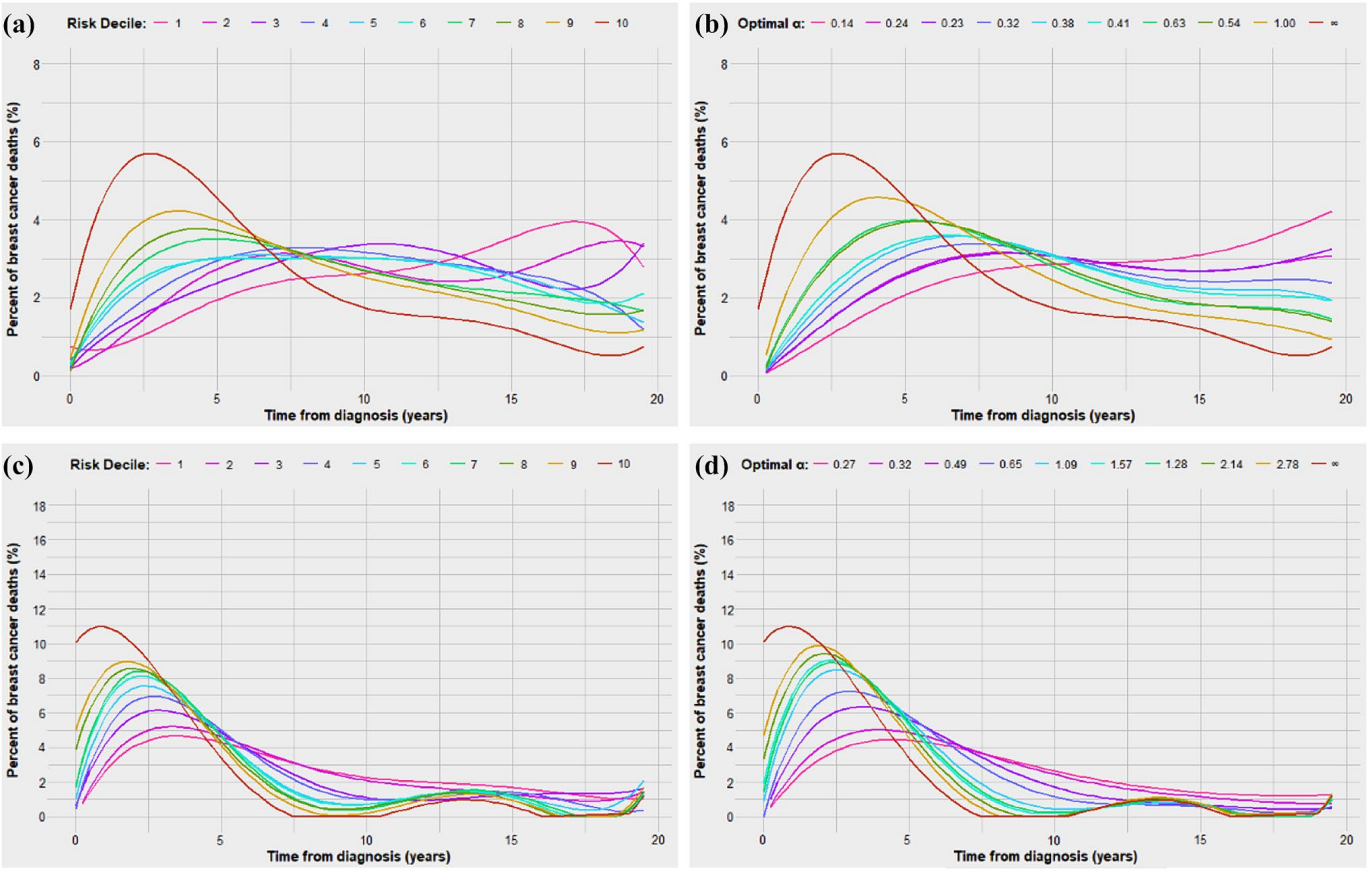
Observed and predicted time-to-death distributions for ER-positive patients (**a**, **b**) and ER-negative patients (**c**, **d**)

We asked if the phenomenon of tumour dormancy was a feature of all breast cancers, of ER-positive breast cancers only or of all breast cancers with low mortality. We divided the 123,705 cases of breast cancer into ER-negative and ER-positive and repeated the steps described above. Fitted values of tumour dormancy by decile for ER-positive and ER-negative patients are presented in eTable 1a and eTable 1b. Observed and modelled time-to-death curves for these subgroups are presented in Fig. 3. Kaplan–Meier survival curves and biannual mortality rates for these subgroups are presented in eFigure 2 and eFigure 3. Independent predictors of time-to-death for ER-specific regression models are presented in eTable 2.

For ER-negative breast cancer patients the curves were similar by decile and the time-to-death did not increase as steeply as for ER-positive patients. The time corresponding to peak mortality rate ranged from 1.5 years in decile 10 to 4.0 years in decile 1. In contrast, for ER-positive breast cancers, the lower risk deciles showed a protracted time-to-death distribution. For women in the highest risk decile, the peak mortality rate was observed at 3.0 years post-diagnosis whereas for women in the three lowest mortality groups, the peak mortality rate time was in excess of 17 years. We estimated the various values of *α* for ER-negative and ER-positive breast cancer for each decile. For women with ER-negative breast cancer, *α* was less than 1.0 only among deciles 1 to 4, suggesting the importance of tumour dormancy in these low risk subgroups. For ER-positive breast cancers, the fitted *α* value ranged from 0.14 for decile 1 to 1.00 for decile 9.

We wished to assess the general applicability of the model for various subgroups categorized by features other than ER status, including nodal status, tumour grade and tumour size. For each of 30 patient subgroups defined by various combinations of these factors, we obtained the observed mortality and we predicted *α* and *c* values using the regression relationships presented in eFigure 1. We calculated the MSE (observed versus predicted) of the normalized mortality rates for all subgroups. The results are summarized in Table 5.

**Table 5:** Observed survival metrics, predicted *α* and *c* value, and fit of dormancy models among predefined patient subgroups

Sub-group	Observed								Predicted		Fit
	Patients (*N*)	Breast cancer deaths (*N*)	Annual death rate (per 100 person-years)	20-year actuarial mortality	Peak mortality time (years from diagnosis)^a^	Median time-to-death (years)	10th percentile time-to-death (years)	90th percentile time-to-death (years)	*α* (reactivations per person-year)^b^	*C* value^b^	MSE std. annual mortality rate (%)^b^
ER-positive	94,944	16,151	1.29	21.6%	4.0	8.0	2.3	16.8	0.49	0.2415	0.5194
* N0*	64,660	6297	0.7	13.0%	5.5	9.3	2.8	17.5	0.32	0.1392	0.3363
Grade I/II	39,176	3029	0.55	10.7%	8.0	10.3	3.3	17.9	0.28	0.1151	0.1774
≤ 2 cm	32,227	2037	0.44	8.9%	9.5	11.1	3.7	18.2	0.24	0.091	0.1414
> 2 cm	6949	992	1.12	19.5%	6.5	9.0	2.8	17.0	0.45	0.2174	0.5144
Grade III/IV	13,490	2025	1.12	18.7%	4.0	7.3	2.4	16.2	0.43	0.2054	0.9935
≤ 2 cm	8800	1032	0.84	14.7%	4.5	8.4	2.8	16.7	0.35	0.1572	0.8238
> 2 cm	4690	993	1.73	26.5%	3.5	6.4	2.2	15.8	0.60	0.3077	1.2271
* N1–N3*	30,058	9729	2.71	39.5%	3.5	7.0	2.1	16.3	0.92	0.5004	0.4392
Grade I/II	14,550	3746	2.02	32.9%	4.9	8.8	2.8	17.1	0.75	0.398	0.21
≤ 2 cm	7728	1406	1.32	24.0%	19.5	10.2	3.3	18.0	0.54	0.2716	0.4506
> 2 cm	6822	2340	2.97	43.4%	4.5	7.8	2.4	16.5	1.03	0.5666	0.2427
Grade III/IV	10,747	4319	3.66	46.8%	3.5	5.6	1.8	14.4	1.14	0.6328	1.2308
≤ 2 cm	3773	1127	2.34	35.4%	4.0	7.2	2.4	16.4	0.81	0.4342	0.5212
> 2 cm	6974	3192	4.58	53.2%	3.0	5.3	1.7	13.7	1.35	0.7593	1.6753
ER-negative	28,761	7502	2.08	28.6%	2.0	3.4	1.1	12.3	1.35	0.3369	1.1056
* N0*	18,057	2891	1.15	18.0%	2.5	4.3	1.5	14.6	0.74	0.1973	0.8652
Grade I/II	4714	662	1.01	16.7%	3.0	6.2	1.8	16.5	0.68	0.1836	1.5207
≤ 2 cm	3575	398	0.77	13.3%	3.5	7.0	2.1	16.5	0.50	0.1424	1.7237
> 2 cm	1139	264	1.89	28.1%	2.5	4.9	1.5	16.8	1.32	0.33	1.8978
Grade III/IV	10,483	1831	1.28	19.2%	2.5	3.8	1.3	13.3	0.81	0.2133	1.1323
≤ 2 cm	5763	782	0.95	15.1%	3.0	4.6	1.8	14.9	0.59	0.163	0.6755
> 2 cm	4720	1049	1.73	24.2%	2.0	3.3	1.2	12.3	1.09	0.2774	1.6708
* N1–N3*	10,582	4538	4.17	46.4%	2.0	2.9	1.0	10.2	2.60	0.6229	0.8036
Grade I/II	2081	792	3.39	42.4%	2.5	4.3	1.4	12.7	2.29	0.552	1.0331
≤ 2 cm	951	284	2.35	33.4%	3.0	5.3	1.8	15.0	1.66	0.4078	2.7702
> 2 cm	1130	508	4.51	50.1%	2.5	3.8	1.3	11.8	2.92	0.6961	0.6459
Grade III/IV	7267	3215	4.44	47.3%	2.0	2.6	1.0	8.4	2.68	0.6412	1.551
≤ 2 cm	2319	816	3.02	37.2%	2.0	3.0	1.2	9.6	1.91	0.465	1.0262
> 2 cm	4948	2399	5.29	52.1%	2.0	2.5	0.9	8.0	3.10	0.7373	1.8366

^a^Peak mortality rate from subgroup specific biannual mortality rate curves

^b^Predictions with ER-specific dormancy models (eFigure 1)

## Discussion

We used a data set of 123,705 breast cancer patients with up to 20 years of follow-up to perform a pattern analysis of time-to-death according to various combinations of prognostic factors. The large sample size and long duration of follow-up accorded us the opportunity to examine mortality rates and times-to-death at a resolution that was not previously possible. In an earlier analysis of the same SEER data set, we showed that a general property of breast cancer patients is that the higher the annual risk of death, the greater the proportion of deaths that occur in the first 5 years [2], e.g. 62% of deaths from grade III breast cancers occur in the first 5 years, only 23% of the deaths from grade I breast cancers occur in the first 5 years.

Using a modelling approach, we ask if the systematic differences observed in the time-to-death and the distinct patterns of the survival curves that we described in 2018 (ref 2) can be accounted for by variation in the duration of an early dormant period. Further, we ask if tumour dormancy is restricted to ER-positive breast cancers. We generated hypothetical survival curves under a simple dormancy model and compared these with the empiric SEER survival curves. We sought to replicate the actual data by incorporating variables which correspond to the probability of metastases being present at diagnosis and the rate of re-activation from tumour dormancy. These two variables, *c* and *α*, respectively, are used successfully to model mortality and time-to-death across 30 patient groupings. Overall, the fit was very good when the two variables were used in combination to model survival differences between the various propensity classes. For ER-negative breast cancers, most values of *α* were relatively large and for these the predicted dormant periods were short. For example, an *α* of 1.0 corresponds to a mean dormant period of one year and values above 1.0 correspond to even shorter dormant periods. Prolonged dormancy was a dominant feature in ER-positive cancer patients and the variation in mortality and time-to-death can be predicted to a large extent by the variation in the rate of tumour reactivation. Thus, it appears that significant variation in tumour dormancy (*α* ≤ 1) is present among low risk ER-negative patients (decile 1 to 4) and all ER-positive patients (deciles 1 to 9).

Here we assume that tumour reactivation is a random event and that within a subgroup, the annual rate of reactivation does not change during the follow-up period. However, between subgroups, the fitted values for rates of reactivation vary markedly; for women in the bottom decile of risk, we estimate the annual reactivation rate to be 14% annually and we expect that 93.9% of cancers will reactivate over 20 years. That is, even if an ER-positive cancer is metastatic at diagnosis, there is a possibility it will not reactivate within 20 years. For women in the top decile of risk, we estimate that almost all cancers will become active within one year.

We performed multivariable quantile regression to determine which variables are most important in predicting the length of tumour dormancy. Strong independent predictors of tumour dormancy included tumour size, grade, nodal status and PR status (Table 4). Race is also important in that black women experience an earlier time-to-death than white women, whereas east Asian women experience a median time-to-death approximately one year later than white women. Interestingly ER-negative status (as compared to ER-positive status) had a median reduction in time-to-death reduction of only 1.7 years; this is less than what we would expect if ER status was the primary driver of tumour dormancy. The adjusted ER effect was smaller than the crude ER effect—this can largely be explained by highly correlated tumour factors within each ER subgroup.

The striking implication of our model is that the lower the mortality rate, the more unpredictable the time-to-death; for example, for women with ER-positive, node-negative, grade I/II breast cancers of less than or equal to 2 cm, the annual mortality rate was almost constant over the five to twenty-year follow-up period (eFigure 4). The interval from 3.7 years to 18.2 years contained 80% of the deaths (Table 5). If a woman has a cancer of this type, her physician may tell her that she has a 8.9% chance of dying of breast cancer in 20 years and that on average, death will occur at 11.1 years. But she is equally as likely to die in year five as in year 20. The prolonged time-to-death of ER-positive breast cancer patients is now well recognized and others have suspected that this is due to tumour dormancy, but have not demonstrated this in a formal way. [1, 6].

There are several strengths to this analysis. We followed the patients for 20 years, and this is ample time to generate characteristic survival curves for various subgroups. It can be seen by inspection of the figures that a five- or ten-year follow up period is insufficient to speculate on the natural history of breast cancer. For example, in evaluating the performance of the BIC score in predicting recurrence in ER-positive breast cancer patients, Sestak et al. define early recurrence as < 5 years and late recurrence as 5–10 years [6]. In our data set, for women with ER-positive breast cancer in the two lowest risk deciles of risk, the peak mortality rate was not reached until 20 years after diagnosis.

There are several weaknesses of our study. Most importantly, the SEER registry does not provide information on tamoxifen or other anti-hormonal therapies. The patients were diagnosed between 1990 and 1999, and we expect a high proportion of the ER-positive breast cancers will have received tamoxifen. Further, tamoxifen is expected to increase the time-to-death [2] but this has not been captured in the current study. Ideally we would present separate mortality curves for women with and without tamoxifen. Future studies should incorporate antihormonal therapy where available.

There is no standard definition of tumour dormancy and most of the literature is based on animal models. In this paper, we interpret tumour dormancy as a state of inactivity, that is the cells in the metastatic niche are not increasing in number and are not generating further metastases. We do not distinguish between no cell division or a balance between cell division and cell death. At present the state of the science does not permit a formal definition of tumour dormancy and this is an area under exploration.

We have modelled our mortality curves under the assumption that, after reactivation, time-to-death is similar for all ten prognostic groups (deciles). This may not be the case; there is little epidemiologic evidence to support or to refute this assumption. In any case, we show that it is not necessary to propose variation in growth rates of various classes of tumour post-reactivation and that the empiric SEER time-to-death curves can be explained by a model which is based entirely on variation in the rate of activation.

Time from metastases to death can be divided into (1) time from metastases to activation, (2) time from activation to distant recurrence, and (3) time from distant recurrence to death. In an ideal situation, we would be able to study time from activation to distance recurrence independent of the other periods, but time of dormancy/activation transition is in-observable. Further the data of first metastatic spread is in-observable and we use the data of diagnosis as a surrogate for this. However, in a recent study using in-house data from our breast cancer follow-up clinic we studied predictors of the time from distant recurrence to death. Interestingly we found no significant predictors of time from distant recurrence to death among 336 ER-positive breast cancer patients. Among ER-negative patients (*N* = 175), a high tumour grade and a short time from diagnosis to distant recurrence were associated with a rapid time-to-death [7].

It has been proposed that chemotherapy would not be effective in treating dormant cancers. If so, we would expect early administration of chemotherapy to have limited value for low risk ER-positive cancer, because most would be in a dormant state for the first few years. In assuming an *α* of 0.63 among a subgroup with predominant chemotherapy use (ER-positive, decile 7), only 27% of cancer cases will emerge from dormancy in the first 6 months from diagnosis. This is the time frame when chemotherapy is given. If chemotherapy were not effective against dormant tumours, we would not expect the benefit of chemotherapy to be present and we would expect the relative benefit to be greater for high-risk ER-positive cancer than for low-risk ER-positive cancers. This is not the case, in the large collaborative study of ER positive breast cancers, the benefit of chemotherapy in terms of hazard ratio was similar for ER-negative and ER-positive cancer and across categories defined by grade and nodal status [8].

In conclusion, we propose that the lower the risk of death from breast cancer the more prolonged is the time-to-death distribution and the more unpredictable the clinical course. We propose that the systematic differences in time-to-death are due to differences in the period of dormancy in the initial course of the cancer. We conclude that median time-to-death is especially prolonged among ER-positive and low-risk ER-negative cancers, and the mean duration of tumour dormancy can be predicted by tumour factors such as grade, tumour size, nodal status and PR status. Large clinical epidemiology studies of women with ER-positive breast cancer may determine whether emergence from dormancy is influenced by host factors and environmental exposures or is a purely random event.

## Electronic supplementary material

Below is the link to the electronic supplementary material.

Supplementary material 1 (DOCX 3302 kb)

## Data Availability

Deidentified patient data is available upon request from the National Cancer Institute—Surveillance, Epidemiology, and End Results Program (https://seer.cancer.gov/seertrack/data/request/). Support for the derived data set is available from the corresponding author SN on request.

## Footnotes

1 Mortality rate distributions are optimal to model tumour dormancy since they are independent of the proportion dead on a given time (unlike time-to-death distributions). However, absolute rates for overall mortality inherently vary with risk and cannot be compared across deciles. For this reason, we compare normalized mortality rate distributions (i.e. cumulative rate at 20 years equals one) to determine an optimal tumour dormancy value.
