# Supplementary material for: A generalizable relationship between mortality and time-to-death among breast cancer patients can be explained by tumour dormancy
Source: Breast Cancer Res Treat. 2019 Jul 1;177(3):691–703. doi: 10.1007/s10549-019-05334-5 (PMC6745044; doi:10.1007/s10549-019-05334-5)

**Supplemental methodology**

**Predicting 20-year risk of breast cancer death**

The assignment of each patient to a risk decile requires a probability of breast cancer death at 20 years of follow-up. We use a Cox model in combination with the Breslow estimator to estimate a baseline hazard for breast cancer-specific mortality. We estimate the probability of breast cancer death for each patient assuming 20 years of follow-up. Categorical predictors include year of diagnosis, patient ethnicity, cancer stage, grade, nodal status, ER status, PR status, radiotherapy and chemotherapy. Continuous variables are modelled as cubic splines and include age at diagnosis, neighbourhood household income and tumour size. Risk deciles are generated by classifying all patients into ten equally sized groups based on their projected 20-year actuarial risk of breast cancer death.

**Modelling tumour dormancy**

We define the tumour reactivation factor (α) to quantify tumour dormancy across subgroups. The tumour reactivation factor represents the rate of reactivation from a dormant state to an active state of a disseminated tumour. Quantitatively it is the rate of tumour reactivation per follow-up year [year^-1^] and can take any value greater than zero.

Since time of cancer dissemination is unobservable it is necessary to model tumour dormancy relative to a reference group. The highest risk decile (decile 10) was used as the reference group. Tumour reactivation rates from 0.01 to 10 by 0.01 and 1,000 (approximating infinity) were applied to the reference group. This was done by simulation for each α value. Patients that died from breast cancer were assigned 240 random numbers (range 0 to 1) for each month of follow-up from 0 to 20 years. If the number was below a threshold value ($1-e^{-\alpha/12}$) the tumour was considered reactivated and the observed time from diagnosis to death began. Time-to-death was redefined as time from diagnosis to tumour reactivation plus observed time from diagnosis to death. Patients with time-to-death exceeding 20 years were censored as alive at 20 years. In each simulation patients were replicated ten times to obtain stabilized distributions of time-to-death.

**Optimal tumour reactivation factor (α)**

We sought to determine what tumour reactivation rate results in a time-to-death distribution that most closely resembles the remaining deciles (decile 1 to 9). However, distributions of time-to-death are inherently dependent on breast cancer mortality. For example, a high-risk decile will have a right skewed distribution because fewer patients are alive in late follow-up. Mortality rate distributions are not skewed because events and person-time are counted among alive patients. Because absolute mortality rates differ across deciles, we normalized biannual mortality rates such that the cumulative mortality rate at 20 years is equal to one. The normalized mortality rate distribution represents an unbiased proportion of breast cancer deaths occurring within a given subinterval. The Mean Square Error (MSE) of the normalized mortality rate distributions were calculated for each *α* and decile combination:

$$MSE_{\alpha,decile}=\frac{1}{40}\times\sum_{i=1}^{40} {({P\left( i \right)}_{\alpha}-P\left( i \right)_{decile})}^{2}$$

Where $i$ is the index corresponding to the biannual time points from 0 to 20 years, and $P(i)$ is the proportion of mortality rate occurring in that time point. MSE values for each *α* and decile combination are presented in eFigure 5. Optimal *α* values are those which minimize the MSE for each decile. Observed and predicted normalized mortality rate distributions for the overall analysis and ER-specific analysis are shown in eFigure 6.

**Multiplication factor (c)**

We aimed to generate predicted annual mortality rates from the normalized mortality rate distribution of the optimal *α* values. To do this we introduce a multiplication factor c (for constant) which when multiplied by the biannual proportions will produce the observed 20-year actuarial breast cancer survival of the corresponding risk decile. That is, for each decile we determine a constant c such that:

$$e^{-\int_{0}^{20} \left( c\times P\left( t \right)_{\hat{\alpha}} \right)dt\times e^{-\int_{0}^{t} \left( c\times P\left( u \right)_{\hat{\alpha}} \right)du}}=S_{obs}(t=20)$$

Where $P\left( t \right)_{\hat{\alpha}}$ is the mortality rate proportion at time $t$ of the optimal *α* normalized mortality rate distribution. $S_{obs}(t=20)$ is the actuarial breast cancer-specific survival at 20 years.

**Biannual mortality rates and distribution**

Observed mortality rates were generated for half-year intervals from 0 to 20 years of follow-up. Observed mortality rates were calculated by dividing the number of breast cancer-specific deaths by the number of follow-up years in the time interval. Predicted mortality rates were generated from the normalized mortality rates of the optimal α value (i.e. predicted normalized mortality rates). This was done by multiplying the normalized rates by the corresponding c value. In both the observed and predicted morality rates a fifth-order polynomial function was fit to generate mortality rate distributions (figure 2e, f and eFigure 2). Peak mortality rate time was outputted from the fitted polynomial.

**Kaplan-Meier survival curves**

Observed Kaplan-Meier survival curves were generated by the standard non-parametric approach. Predicted Kaplan-Meier survival curves were generated by applying the predicted biannual mortality rates over a 20-year follow-up period.

**Time-to-death distribution**

We aimed to generate a distribution of time-to-death among patients that die from breast cancer. Selecting time-to-death among patients that die from breast cancer will result in a bias distribution because of competing risks of death and censoring. To overcome this we derive a time-to-death distribution from biannual mortality rates directly. The proportion of breast cancer deaths occurring between time $t$ and $t+dt$ is equal to:

$$P\left( t,t+dt \right)=\frac{e^{-\int_{t}^{t+dt} (r(t)\times dt)}\times e^{-\int_{0}^{t} (r(u)\times du)}}{e^{-\int_{0}^{20} (r(t)\times dt)}\times e^{-\int_{0}^{t} (r(u)\times du)}}$$

Where $e^{-\int_{t}^{t+dt} (r(t)\times dt)}$ in the numerator corresponds to the change in survival between times $t$ and $t+dt$, and $e^{-\int_{0}^{t} (r(u)\times du)}$ is the survival up to time $t$. The latter term is necessary in order to account for the declining number of alive patients in the follow-up. For example, a high-risk cancer population will have a small proportion of deaths occurring in years 15 to 20 because most women will have died from breast cancer between years 0 to 15. A time-to-death distribution approximates a normalized morality rate distribution when the overall survival is high (i.e. $e^{-\int_{0}^{t} (r(u)\times du)}\cong1$). The denominator of the equation above is necessary in order to standardize into a proportion (i.e. sum of values between year 0 and 20 equals one). Time-to-death biannual values were fit using a fifth-order polynomial function (figure 2a, b) to generate time-to-death distributions.

**Quantile regression models**

Quantile regression was used to determine independent predictors of median time-to-death among women that died from breast cancer. A major consideration with this approach is the potential of bias effect as a result of spurious associations with competing risks of death and censoring. For example, older women that died from breast cancer will have a time-to-death distribution that is over-represented in years following diagnosis (right skewed) because of competing rates of death. To overcome this bias we restricted our cohort to women diagnosed age 80 or younger, and accounted for the probability of being censored as a result of other causes of death or loss to follow-up. Probability of being censored in 20 years was estimated for each patient using a Cox model described previously. The model outcome was non-breast cancer-specific deaths or any loss to follow-up occurring in the first 20 years of follow-up. To ensure patients were able to have 20 years of follow-up we restricted our cohort to breast cancer diagnosis occurring between 1990-1994. In total there were 48,281 eligible subjects in the sub-analysis. In the first 20 years of follow-up, 32.1% of the patients had died from non-breast cancer-specific or unknown causes and 2.3% were lost to follow-up. Stabilized inverse probability censor weights (IPCWs) were calculated for each patient using the output probability.^1^ Stabilized weights ranged from 0.34 to 14.60 among the highest to lowest likelihood of censoring subjects respectively. Weights were then incorporated in the quantile regression model such that individuals more likely to be censored were weighted less than individuals less likely to be censored. The intercept value is interpreted as the median time-to-death (in years) for patients in the low risk reference category (white ethnicity, size <1cm, Grade I, node negative ER positive, PR positive, no radiotherapy, No/Unknown chemotherapy). Effect estimates are interpreted as the change in median time-to-death of a given value relative to the reference level. Positive values indicate an increase in median time-to-death while negative values indicate a decrease in median time-to-death.

**Supplemental tables**

**eTable 1a:** Factors related to breast cancer mortality and time-to-death in risk subgroups (deciles). ER-positive only.

| Risk Decile | Number of patients (N) | Annual death rate (per 100 person-years) | 20-year actuarial mortality (%) | Peak mortality time (years)* | Median time-to-death (years) | 10th percentile time-to-death (years) | 90th percentile time-to-death (years) | α (reactivations per person-year) | C value |
| --- | --- | --- | --- | --- | --- | --- | --- | --- | --- |
| 1 | 9,494 | 0.23 | 4.9% | 17.5 | 12.7 | 4.7 | 18.4 | 0.14 | 0.0503 |
| 2 | 9,494 | 0.38 | 7.9% | 19.5 | 11.3 | 4.4 | 18.7 | 0.24 | 0.0818 |
| 3 | 9,495 | 0.51 | 10.1% | 19.5 | 11.0 | 4.2 | 18.1 | 0.23 | 0.1060 |
| 4 | 9,494 | 0.65 | 12.2% | 9.5 | 10.3 | 3.5 | 17.5 | 0.32 | 0.1303 |
| 5 | 9,495 | 0.75 | 14.0% | 8.0 | 9.8 | 3.2 | 17.1 | 0.38 | 0.1507 |
| 6 | 9,494 | 1.02 | 18.7% | 11.5 | 10.0 | 3.2 | 17.7 | 0.41 | 0.2068 |
| 7 | 9,495 | 1.28 | 22.3% | 5.5 | 9.0 | 2.9 | 17.6 | 0.63 | 0.2522 |
| 8 | 9,494 | 1.84 | 30.3% | 5.0 | 8.4 | 2.8 | 16.9 | 0.54 | 0.3603 |
| 9 | 9,495 | 2.78 | 40.7% | 4.0 | 7.4 | 2.3 | 16.1 | 1.00 | 0.5222 |
| 10 | 9,494 | 5.72 | 62.2% | 3.0 | 5.4 | 1.6 | 14.2 | InF (Reference) | 0.9735 |

*Peak mortality rate from biannual mortality rate curves (eFigure 2a).

**eTable 1b:** Factors related to breast cancer mortality and time-to-death in risk subgroups (deciles). ER-negative only.

| Risk Decile | Number of patients (N) | Annual death rate (per 100 person-years) | 20-year actuarial mortality (%) | Peak mortality time (years)* | Median time-to-death (years) | 10th percentile time-to-death (years) | 90th percentile time-to-death (years) | α (reactivations per person-year) | C value |
| --- | --- | --- | --- | --- | --- | --- | --- | --- | --- |
| 1 | 2,876 | 0.58 | 10.2% | 4.0 | 7.0 | 2.5 | 15.6 | 0.27 | 0.1076 |
| 2 | 2,876 | 0.77 | 13.1% | 3.0 | 6.2 | 2.2 | 15.8 | 0.32 | 0.1400 |
| 3 | 2,876 | 0.91 | 15.4% | 3.0 | 5.3 | 1.8 | 16.6 | 0.49 | 0.1666 |
| 4 | 2,876 | 1.17 | 17.9% | 2.5 | 4.6 | 1.8 | 13.8 | 0.65 | 0.1977 |
| 5 | 2,876 | 1.43 | 21.7% | 2.0 | 4.1 | 1.5 | 14.9 | 1.09 | 0.2443 |
| 6 | 2,877 | 1.75 | 25.2% | 2.0 | 3.8 | 1.4 | 12.3 | 1.57 | 0.2907 |
| 7 | 2,876 | 2.20 | 30.2% | 2.0 | 3.6 | 1.4 | 13.0 | 1.28 | 0.3596 |
| 8 | 2,876 | 2.94 | 36.7% | 2.0 | 3.2 | 1.2 | 11.3 | 2.14 | 0.4565 |
| 9 | 2,876 | 4.82 | 50.9% | 2.0 | 3.0 | 1.1 | 11.3 | 2.78 | 0.7122 |
| 10 | 2,876 | 9.58 | 68.6% | 1.5 | 2.2 | 0.8 | 6.4 | InF (Reference) | 1.1570 |

*Peak mortality rate from biannual mortality rate curves (eFigure 2c).

**eTable 2:** Predictors of time-to-death for ER-positive and ER-negative patients.

| **Predictor** | **Value** | **ER-positive** | | | | **ER-negative** | | | |
| --- | --- | --- | --- | --- | --- | --- | --- | --- | --- |
|  |  | Unadjusted | | Adjusted | | Unadjusted | | Adjusted | |
|  |  | Median time-to-death (years) | Difference* (years) | Difference (years)** | P | Median time-to-death (years) | Difference* (years) | Difference (years)** | P |
| ***Overall*** |  | 7.08 |  | 13.25*** | <.0001 | 3.50 |  | 12.31*** | <.0001 |
| Year of diagnosis | 1990 | 6.67 |  | Reference |  | 3.25 |  | Reference |  |
|  | 1991 | 6.83 | 0.17 | 0.32 | 0.2736 | 3.33 | 0.08 | 0.08 | 0.6865 |
|  | 1992 | 6.92 | 0.25 | -0.02 | 0.9436 | 3.58 | 0.33 | 0.34 | 0.09 |
|  | 1993 | 7.17 | 0.50 | 0.21 | 0.4427 | 3.58 | 0.33 | 0.42 | 0.0374 |
|  | 1994 | 7.75 | 1.08 | 0.65 | 0.0352 | 3.67 | 0.42 | 0.28 | 0.1838 |
| Age at diagnosis | <50 | 7.17 |  | Reference |  | 3.58 |  | Reference |  |
|  | 50-59 | 7.33 | 0.17 | 0.15 | 0.4714 | 3.42 | -0.17 | -0.13 | 0.3365 |
|  | 60-69 | 7.50 | 0.33 | -0.13 | 0.5277 | 3.75 | 0.17 | -0.02 | 0.8919 |
|  | 70-80 | 5.75 | -1.42 | -1.49 | <.0001 | 3.08 | -0.50 | -0.40 | 0.0547 |
| Ethnicity | White | 7.17 |  | Reference |  | 3.58 |  | Reference |  |
|  | Black | 5.83 | -1.33 | -0.54 | 0.0316 | 3.08 | -0.50 | -0.19 | 0.1655 |
|  | East Asian | 8.5 | 1.33 | 1.28 | 0.0174 | 4.33 | 0.75 | 0.97 | 0.0194 |
|  | Southeast Asian | 7.00 | -0.17 | 0.09 | 0.8161 | 4.25 | 0.67 | 0.13 | 0.773 |
|  | Other/Unknown | 6.67 | -0.50 | -0.16 | 0.8451 | 4.08 | 0.50 | 0.78 | 0.2176 |
| Tumour size (cm) | <1cm | 10.17 |  | Reference |  | 5.08 |  | Reference |  |
|  | 1-2cm | 8.58 | -1.58 | -0.84 | 0.1326 | 4.58 | -0.50 | -0.15 | 0.7292 |
|  | 2-3cm | 6.83 | -3.33 | -1.87 | 0.0008 | 3.58 | -1.50 | -0.70 | 0.0925 |
|  | 3-5cm | 6.17 | -4.00 | -2.06 | 0.0002 | 3.00 | -2.08 | -1.08 | 0.0055 |
|  | 5+cm | 5.08 | -5.08 | -2.54 | <.0001 | 2.42 | -2.67 | -1.40 | 0.0005 |
| Tumour grade | I | 11.42 |  | Reference |  | 10.58 |  | Reference |  |
|  | II | 8.25 | -3.17 | -2.46 | <.0001 | 4.92 | -5.67 | -5.23 | <.0001 |
|  | III | 5.67 | -5.75 | -4.52 | <.0001 | 3.00 | -7.58 | -7.01 | <.0001 |
|  | IV | 5.50 | -5.92 | -5.09 | <.0001 | 2.67 | -7.92 | -7.34 | <.0001 |
|  | Unknown | 7.50 | -3.92 | -2.97 | <.0001 | 4.33 | -6.25 | -5.92 | <.0001 |
| Nodal status | N0 | 8.50 |  | Reference |  | 4.33 |  | Reference |  |
|  | N1 | 7.17 | -1.33 | -0.82 | 0.0007 | 3.33 | -1.00 | -0.56 | 0.0001 |
|  | N2 | 6.25 | -2.25 | -1.53 | <.0001 | 2.92 | -1.42 | -0.90 | <.0001 |
|  | N3 | 4.75 | -3.75 | -2.69 | <.0001 | 2.33 | -2.00 | -1.54 | <.0001 |
|  | Unknown | 3.42 | -5.08 | -3.17 | <.0001 | 1.50 | -2.83 | -1.85 | <.0001 |
| PR status | Positive | 7.58 |  | Reference |  | 5.00 |  | Reference |  |
|  | Negative | 5.33 | -2.25 | -1.75 | <.0001 | 3.33 | -1.67 | -1.36 | <.0001 |
|  | Unknown | 6.58 | -1.00 | -1.18 | 0.0195 | 3.50 | -1.50 | -1.15 | 0.0249 |
| Radiotherapy | No | 6.83 |  | Reference |  | 3.42 |  | Reference |  |
|  | Yes | 7.58 | 0.75 | 0.41 | 0.0159 | 3.75 | 0.33 | 0.15 | 0.2811 |
|  | Unknown | 6.00 | -0.83 | -0.43 | 0.3306 | 3.17 | -0.25 | -0.44 | 0.2534 |
| Chemotherapy | No/Unknown | 7.83 |  | Reference |  | 3.92 |  | Reference |  |
|  | Yes | 6.67 | -1.17 | -0.28 | 0.1783 | 3.33 | -0.58 | -0.08 | 0.5633 |

*Difference in median time-to-death relative to reference value.

**Independent difference in median time-to-death after adjusting for all covariates in table.

***Median time-to-death of the reference group; diagnosed in year 1990, age <50, white ethnicity, <1cm, grade I, N0, PR-positive, no radiotherapy, no/unknown chemotherapy.

**Supplemental figures**

**eFigure 1:** Relationship between actuarial probability of breast cancer death at 20 years versus c value (left), and c value versus optimal tumour dormancy factor (α) (right). Modelled relationships for all patients (a, b), ER-positive patients (c, d) and ER-negative patients (e, f).

**d)**

**f)**

**e)**

**c)**

**b)**

**a)**

**
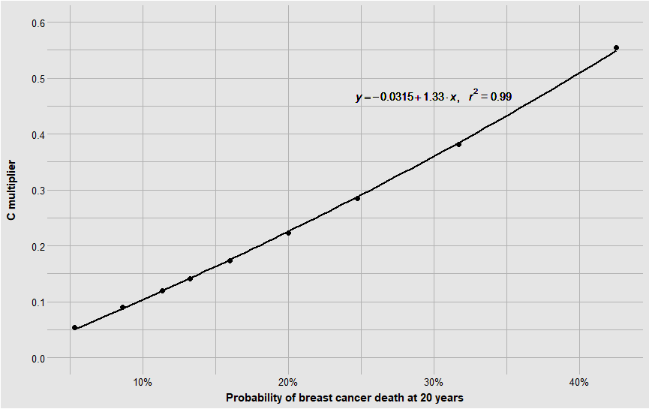

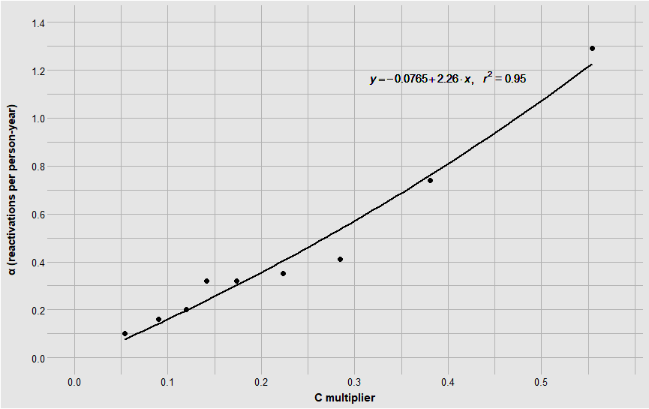
**


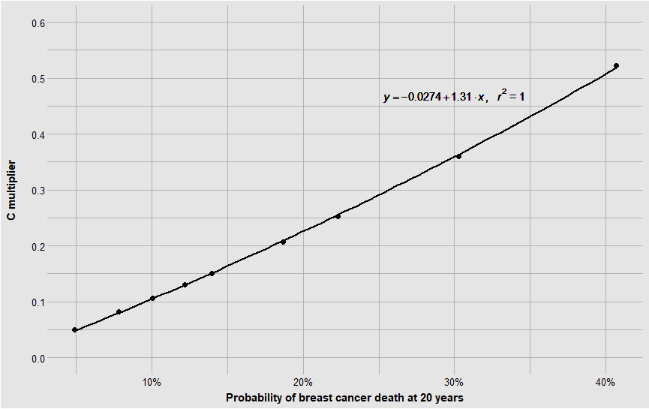

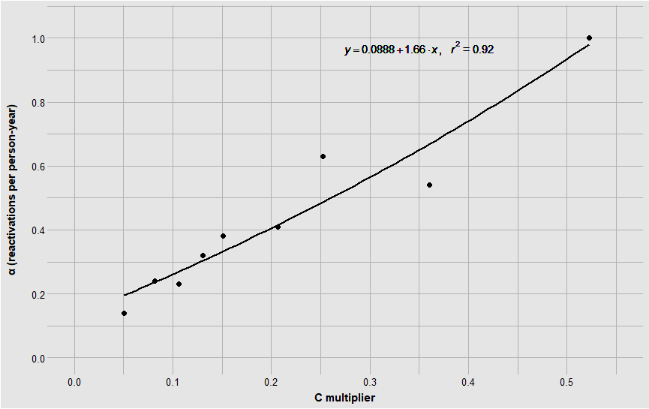


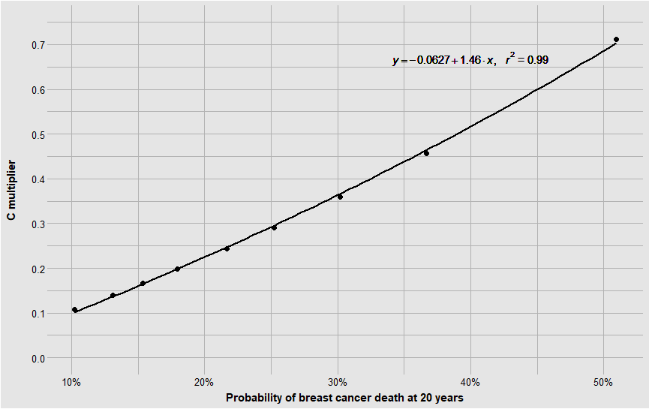

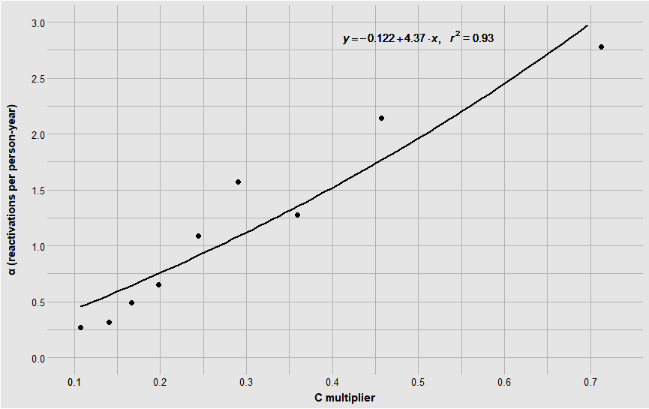


**eFigure 2:** Observed and predicted biannual mortality rates and distributions for ER-positive patients (a, b) and ER-negative patients (c, d).

**a)**

**b)**


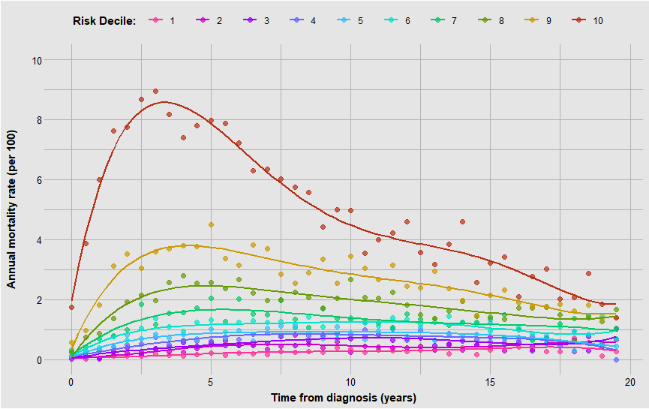

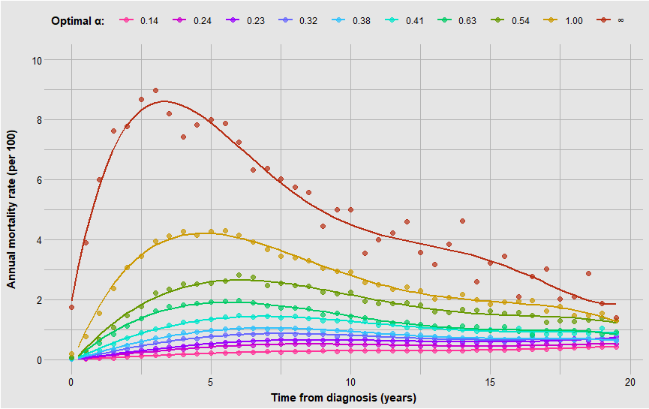


**c)**

**d)**


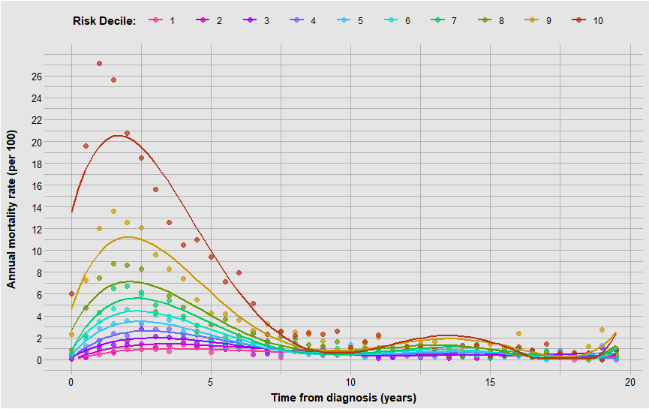

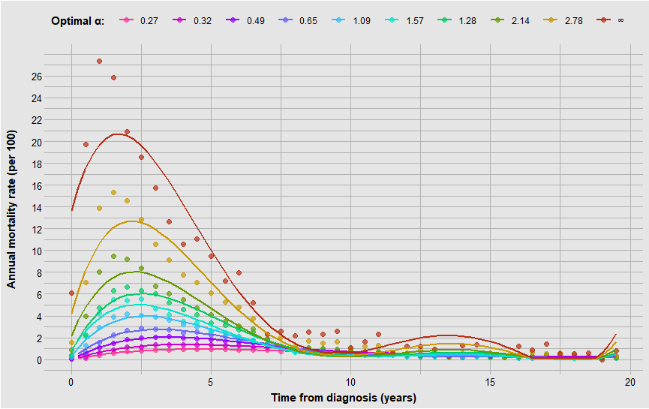


**eFigure 3:** Observed and predicted Kaplan-Meier survival curves for ER-positive patients (a, b) and ER-negative patients (c, d).

**a)**

**b)**

**c)**


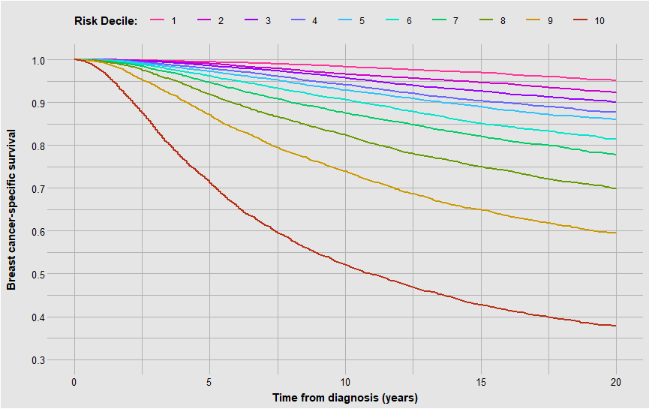

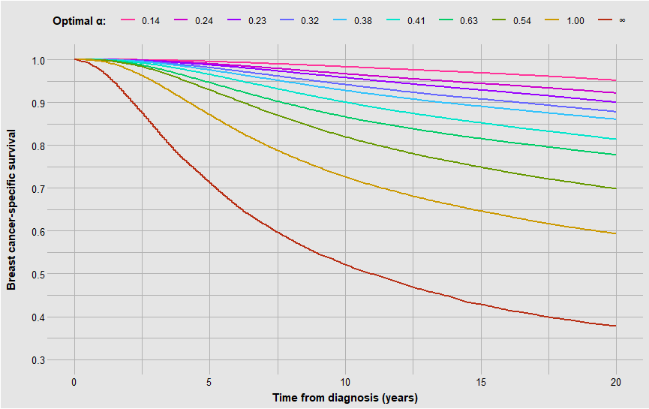


**d)**


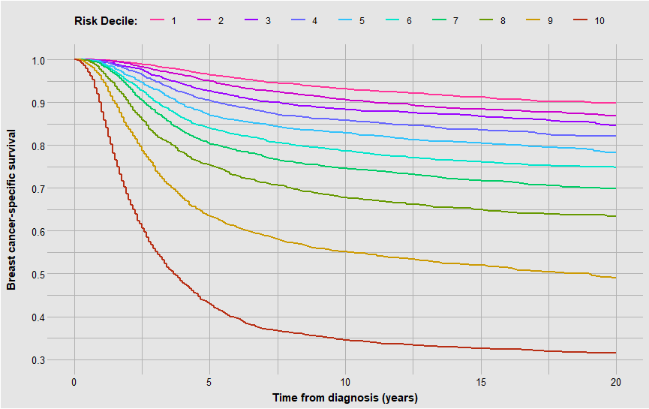

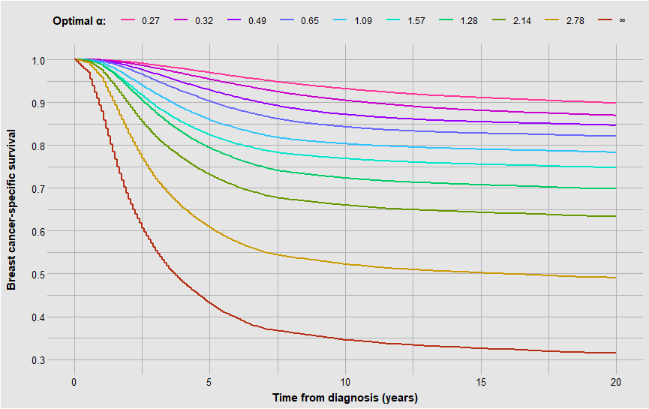


**eFigure 4:** Observed and predicted biannual mortality rates and distributions among breast cancer patients with ER-positive, node negative, grade I/II tumour ≤ 2cm.


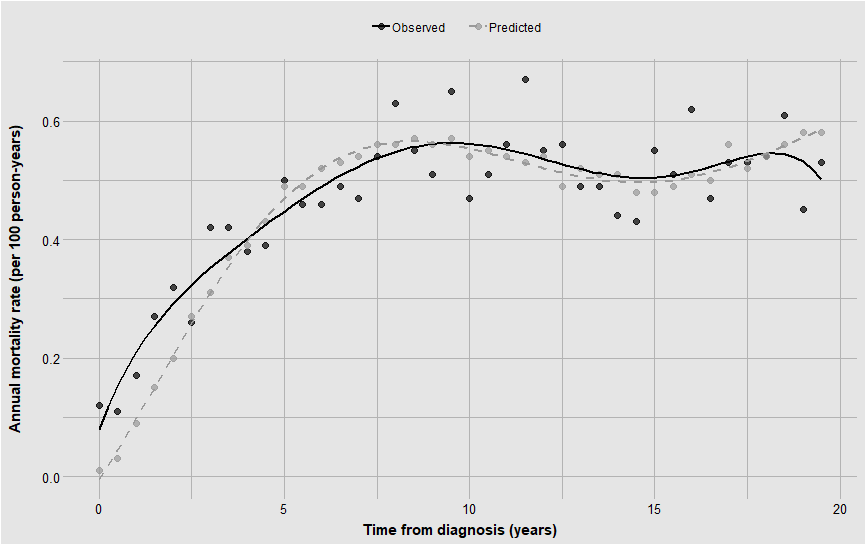


_____________________________________________________________________________

**Legend: Predicted values for biannual mortality rates based on reference distribution (ER-positive decile 10) after applying a tumour dormancy value (α = 0.24) and multiplication factor (C value = 0.091). The optimal c value and α is regressed (eFigure 1c, d) using the 20-year actuarial breast cancer mortality of the patient subgroup (ER-positive, node negative, grade I/II, tumour size ≤ 2cm).*

**eFigure 5:** Mean Square Error (MSE) of normalized mortality rate distribution with varying α values for each patient decile. Minimum MSE for each decile corresponds to optimal α. Optimization results presented on a linear x-scale (left) and natural logarithm x-scale (right). Optimization results for all patients (a, b), ER-positive patients (c, d) and ER-negative patients (e, f).

**f)**

**e)**

**c)**

**b)**

**a)**


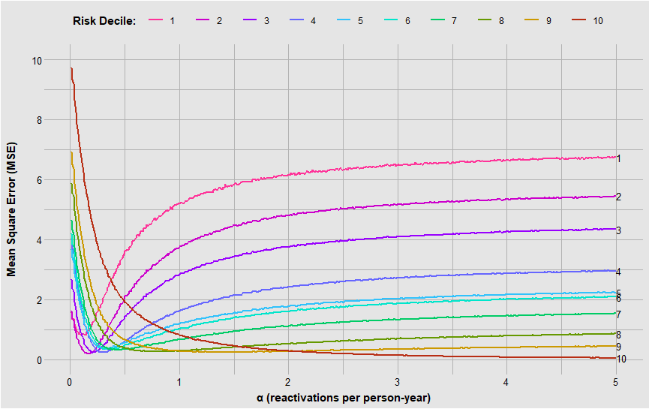

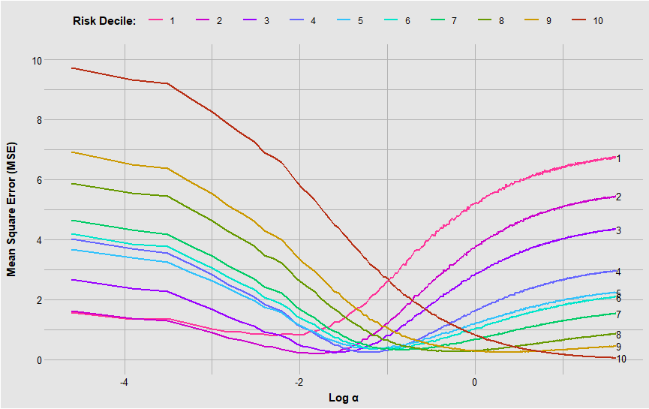


**d)**


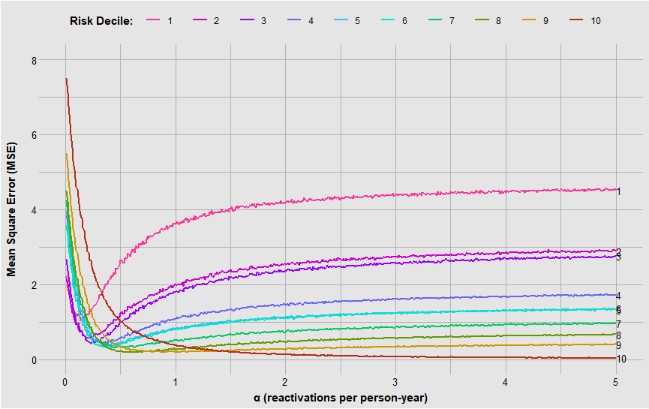

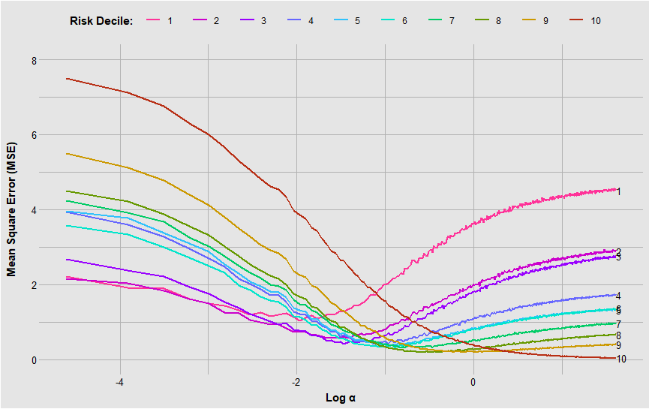


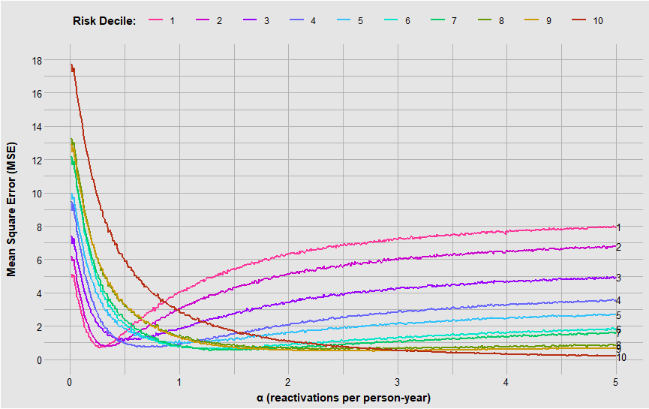

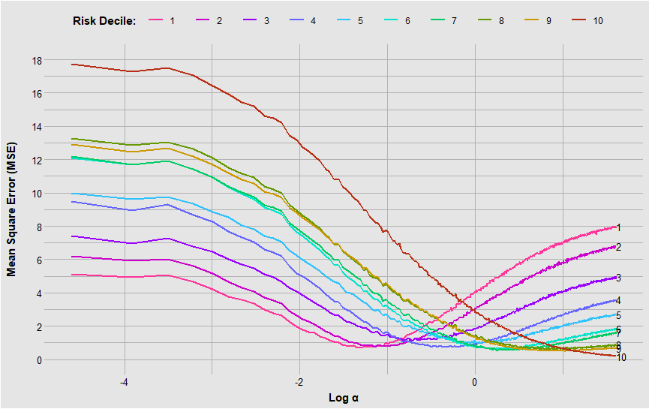


**eFigure 6:** Observed and predicted normalized mortality rate distributions using optimal α. Distributions for each decile among all patients (a, b), ER-positive patients (c, d) and ER-negative patients (e, f).

**f)**

**e)**

**c)**

**b)**

**a)**


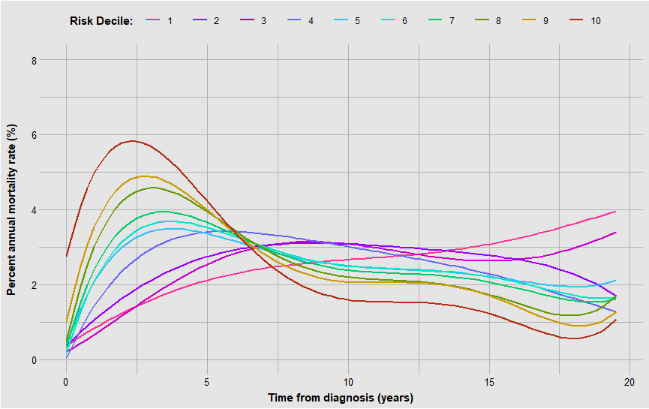

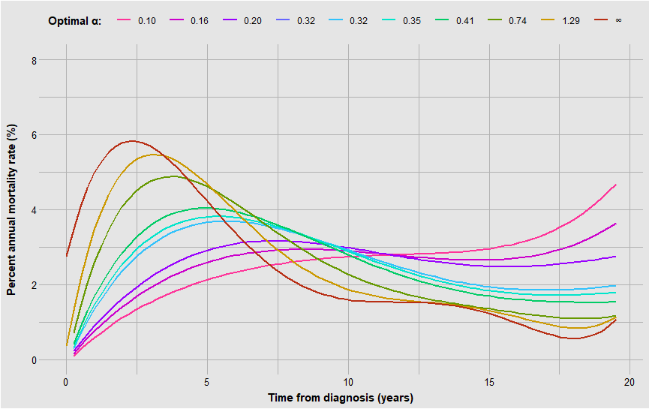


**d)**


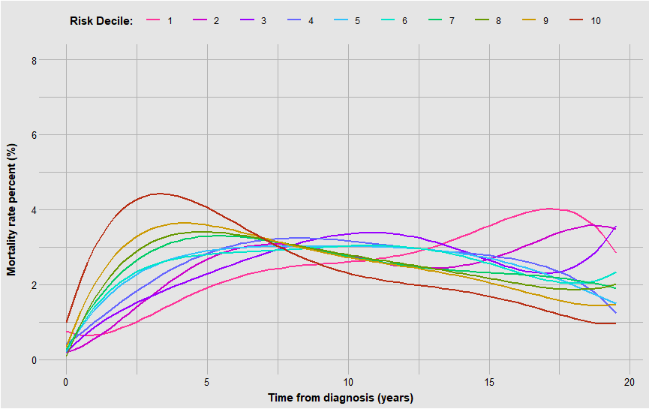

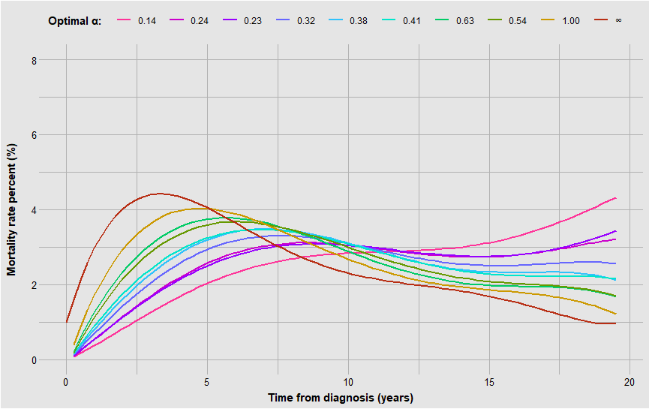


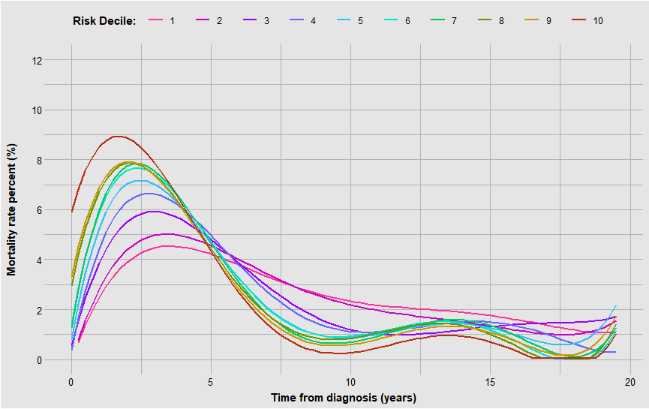

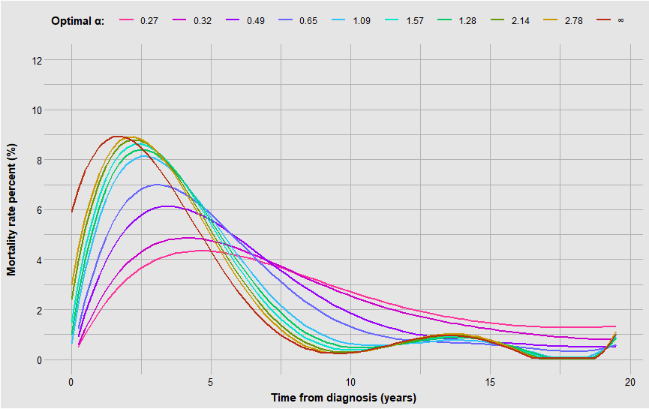

Supplement: Supplementary file 1 — Supplementary material 1 (DOCX 3302 kb) [file 10549_2019_5334_MOESM1_ESM.docx]
